# *Prevotellaceae* Modulates Colorectal Cancer Immune Microenvironment to Assist Anti-PD-L1 Immunotherapy

**DOI:** 10.5152/tjg.2024.23683

**Published:** 2024-12-01

**Authors:** Song Xu, Jianqiao Kong, Yang Dai, Hengping Li

**Affiliations:** Department of General Surgery, Xiangyang No.1 People’s Hospital, Hubei University of Medicine, Xiangyang, China

**Keywords:** *Prevotellaceae*, colorectal cancer, immune microenvironment, anti-PD-L1, immunotherapy

## Abstract

**Background/Aims:**

Colorectal cancer (CRC) stands as the third most prevalent cancer on a global scale. In recent years, immunotherapy, such as anti-PD-L1 treatment, has demonstrated promising therapeutic outcomes in CRC. However, studies have suggested that intestinal microbiota may influence the efficacy of anti-PD-L1 immunotherapy. This study aimed to investigate the linkage between intestinal bacteria and anti-PD-L1 therapy.

**Materials and Methods:**

Bioinformatics analysis was employed to study the correlation between the intestinal microbiota of CRC patients and immune infiltration. The study delved into the relationship between *Prevotellaceae* and immune-related genes in CRC. Mouse experiments were conducted to validate the association between *Prevotellaceae* abundance and the efficacy of anti-PD-L1 tumor treatment. *Prevotellaceae* abundance in mouse feces was assayed by 16S sequencing. Flow cytometry was utilized to assay immune cell infiltration in patient tumor tissues, while western blot and quantitative polymerase chain reaction (qPCR) assays measured *IFN-γ*, *IL-2*, and *PD-L1* levels in tumor tissues.

**Results:**

The high immune cell infiltration group demonstrated reduced tumor purity when compared with the group displaying low immune cell infiltration. Substantial variances were discerned in the Stromal Score, Immune Score, ESTIMATE Score, and Tumor Purity among the 3 distinct subtypes. The community evenness in the gut microbiota of CRC patients from cluster 2 and cluster 3 subtypes displayed significant differences. Members of the *Prevotellaceae* family were significantly enriched in the gut microbiota of cluster 3 subtype patients. *In vivo* experiments ascertained the supportive role of *Prevotellaceae* in anti-PD-L1 immunotherapy.

**Conclusion:**

The facilitating effect of *Prevotellaceae* on anti-PD-L1 treatment was demonstrated in CRC. The findings suggest that elevating *Prevotellaceae* abundance may offer a new direction for assisting in CRC immunotherapy and provide a foundation for devising more effective CRC immunotherapeutic strategies.

**Main Points**

- For the first time, *Prevotellaceae* was found to assist anti-PD-L1 immunotherapy for CRC.
- *Prevotellaceae* assisted anti-PD-L1 immunotherapy by regulating the immune microenvironment of CRC.
- The findings of our study demonstrated for the first time that *Prevotellaceae* abundance influenced the efficacy of CRC anti-PD-L1 immunotherapy.
- The findings confirm that a high abundance of *Prevotellaceae* enhanced the efficacy of anti-PD-L1 tumor therapy.

## Introduction

Colorectal cancer (CRC) continues to hold a prominent position as a leading contributor to cancer-related morbidity and mortality on a global scale. It occupies the third position in terms of incidence and the second position in terms of mortality.^1^ In developing countries like Argentina, Brazil, and China, there has been a notable surge of approximately 20% in the rates of both CRC incidence and mortality.^2^ Research has shown that several immunotherapeutic agents, including nivolumab and pembrolizumab, have received Food and Drug Administration (FDA) approval for cancer treatment.^3^ Immune checkpoint inhibitors (ICIs) targeting programmed cell death protein 1 (PD-1) or its ligand 1 (PD-L1) have achieved significant clinical success in anticancer therapy.^4^ For instance, BMS-1, a small molecule inhibitor of the PD-1/PD-L1 interaction, exerts similar effects to PD-1/PD-L1 antibodies, demonstrating its potential to replace antibodies in immunotherapy.^5^ Nonetheless, PD-1/PD-L1 inhibitors have the potential to elicit a spectrum of immune-related adverse events.^6^ Therefore, the quest for novel therapeutic approaches to assist in anti-PD-L1 immunotherapy for CRC is of utmost importance.

Based on the etiology, approximately 20% of CRC patients have a family history of CRC, while others present with sporadic CRC. Epidemiological research has shown that the onset and progression of CRC represent a multifaceted and multistep process influenced by environmental and genetic factors.^7^ Inflammation is associated with tumor progression,^8^ and alterations in the gut microbiota play a crucial part in inflammatory responses.^9,10^ Metabolites of the gut microbiota, such as short-chain fatty acids (SCFAs), including butyrate, possess anti-inflammatory properties.^11^ Butyrate and its derivative phenylalanine-butyramide can prevent doxorubicin-induced cardiotoxicity,^12,13^ and a reduction in the abundance of microbes capable of producing butyrate has been found to be involved in the pathogenesis of doxorubicin-related cardiotoxicity.^12^ The gut microbiota *Prevotellaceae* family is capable of producing butyrate, and long-term treatment with nicotinamide mononucleotide (NMN) helps maintain gut homeostasis by modulating the gut microbiota.^14^ Furthermore, fecal transplantation can enhance the efficacy of PD-1/PD-L1 inhibitors in cancer therapy.^15^ For instance, in melanoma patients undergoing immunotherapy, higher dietary fiber intake is significantly linked with improved progression-free survival among the 128 individuals receiving immune checkpoint blockade (ICB), with the most pronounced benefits observed in individuals with adequate dietary fiber intake and without probiotic use.^16^ However, it remains unclear whether manipulating gut microbiota abundance can assist in PD-1/PD-L1 inhibitor therapy for CRC. Therefore, the study delved deeper into whether gut microbiota abundance can aid anti-PD-L1 immunotherapy, with the hope of providing new insights into the treatment of CRC.

In this study, we initiated our investigation with extensive bioinformatics analysis, revealing a significant enrichment of members from the *Prevotellaceae* family in the gut microbiota of cluster 3 subtype CRC individuals. We also observed a positive correlation between higher *Prevotellaceae* abundance and increased immune infiltration in CRC. Subsequently, through *in vivo* experiments and cellular functional assays, we verified that a high abundance of *Prevotellaceae* fostered the efficacy of anti-PD-L1 tumor therapy. Our research findings confirmed the role of *Prevotellaceae* in the process of anti-PD-L1 immunotherapy, thereby offering a novel avenue for enhancing the effectiveness of immunotherapy in treating CRC.

## Materials and Methods

### Bioinformatics

Thirty-three CRC patient RNAseq expression matrices were obtained from the Zenodo database (https://doi.org/10.5281/zenodo.2604777). The gene set variation analysis (GSVA) package was utilized to perform single-sample Gene Set Enrichment Analysis (ssGSEA) analysis on the CRC patient samples. Based on ssGSEA results, the ConsensusClusterPlus package was employed to perform K-means consensus clustering, categorizing the samples into 3 distinct immune subgroups. Data from 16S amplicon sequencing, available in the SRP117763 project, were supplied by the NCBI’s (National Center for Biotechnology Information) sequence read archive (SRA) database (https://www.ncbi.nlm.nih.gov/sra/). Following the download, the data in SRA format were converted to FastQ format using the “SRA Toolkit” software package. Sample data were processed using the q2-dada2 plugin within QIIME2 (version 2021.4, https://docs.qiime2.org/2021.4/). A phylogenetic tree was constructed utilizing the q2-fragment-insertion plugin (https://github.com/qiime2/q2-fragment-insertion). Reference sequences clustered at 99% similarity were downloaded from the Greengenes database (http://greengenes.secondgenome.com), specifically the V3-V4 region (341F/805R) in the 99_otus.fasta file. These sequences were used to train a naive Bayes classifier for bacterial species annotation within this study, facilitated by the q2-feature-classifier plugin (https://github.com/qiime2/q2-feature-classifier). The q2-diversity plugin (https://github.com/qiime2/q2-diversity) was harnessed to conduct α-diversity and β-diversity analyses among different immune subgroups, thereby identifying the composition of gut microbiota species and differential microbial taxa in distinct immune subtypes of CRC patients. Pearson correlation analysis was utilized to assess the relationship between gut microbiota abundance and immune components, as well as immune-related gene sets determined in ssGSEA. This analysis aimed to identify bacterial strains within the gut microbiota that were associated with immune functions. The relationship between *Prevotellaceae* abundance and immune-related genes in CRC was examined. Samples were categorized into high and low *Prevotellaceae* abundance groups, and differential expression analysis was done utilizing the edgeR package with predefined criteria (|logFC| > 1.5, padj < 0.05). Subsequently, cluster 1 and cluster 2 were merged into subclass A, with cluster 3 designated as subclass B. Differential gene expression analysis was done utilizing the edgeR package between subclass A and subclass B patients. The overlapping differentially expressed genes (DEGs) were identified, providing insight into genes associated with both *Prevotellaceae* and the immune system.

### Animal Experiments

All animal experiments were executed in strict compliance with the “Guide for the Care and Use of Laboratory Animals” and received approval from the Animal Care and Use Committee of Xiangyang No.1 People’s Hospital (approval number: XYYYE20240003, date: 12/18/2023). The CRC mouse models were established using the azoxymethane (AOM)/dextran sulfate sodium (DSS) regime, with specific procedures detailed in this literature.^17^

Twenty male C57BL/6 mice (aged 5-6 weeks) were acquired for the study. At weeks 2, 5, and 8, the mice received intraperitoneal injections of 7.4 mg/kg AOM, followed by 3 cycles of 3% DSS administered in drinking water for a continuous 7-day period. The mice were stratified into 4 groups, each containing 5 mice, and co-housed to minimize potential cage effects.

From week 6 until the endpoint, the control group received bi-weekly intraperitoneal injections of anti-mouse IgG1 isotype control antibody (Thermo Fisher Scientific, USA) and were orally administered phosphate-buffered saline (1 g/kg/day) for one week, totaling 10 injections of anti-mouse IgG1 isotype control antibody. The experimental group received semi-weekly intraperitoneal injections of anti-mouse PD-L1 antibody (Thermo Fisher Scientific, USA) for 10 injections. Lyophilized *Prevotellaceae* (*P. loescheii*) (ATCC, USA) were resuspended in phosphate-buffered saline at 5 × 109 colony-forming units (CFUs)/mL, and the mice were orally administered the *P. loescheii* solution (1 g/kg/day) for 1 week. Additionally, the experimental group received both semi-weekly intraperitoneal injections of anti-mouse PD-L1 antibody and oral administration of the *P. loescheii* solution (1 g/kg/day) for 1 week, resulting in a total of 10 injections of anti-mouse PD-L1 antibody.

After a 2-week interval post-treatment, all mice were humanely euthanized, and their colons were surgically excised. The colons were longitudinally opened, and luminal contents were washed with PBS. Subsequently, the intestines were flattened on filter paper and fixed overnight in 10% phosphate-buffered formalin. Tumor numbers within the small and large intestines were quantified using an inverted microscope. Prior to euthanasia, fresh fecal pellets were collected from the treated mice and immediately frozen in liquid nitrogen for subsequent DNA extraction and 16S sequencing analysis.

### Quantitative Reverse Transcription Polymerase Chain Reaction (qRT-PCR)

Total RNA was isolated from tissue samples utilizing TRIzol reagent (Invitrogen, USA). Subsequently, 1 μg of total RNA was reverse transcribed into cDNA using the PrimeScript RT kit (Takara, Japan). Quantitative reverse transcription polymerase chain reaction (qRT-PCR) assays were executed on the Applied Biosystems™ 7500 Real-Time PCR System (Thermo Fisher Scientific, USA) using TB Green Premix Ex Taq II (Takara, Japan). *GAPDH* served as the reference gene. Each sample was assayed in triplicate, and each assay was replicated three times. The relative mRNA expression levels were analyzed by harnessing the 2^−ΔΔCt^ method. Table 1 shows the primer sequences.

### Flow Cytometry

Tumor tissue from mice was digested into a single-cell suspension. Cells were then kept with antibodies, including PerCP-Cy5.5 anti-mouse CD3 Antibody, PE anti-mouse CD4 Antibody, and APC anti-mouse CD8 Antibody (Biolegend, USA), at room temperature for 1 hour. Afterward, cells were subjected to 2 consecutive washes with 4 mL of buffer, followed by centrifugation, and then resuspended in 0.5 mL of running buffer for analysis. Flow cytometry analysis was conducted utilizing the NovoCyte flow cytometer system (Agilent, USA), with data analysis being carried out through the utilization of FlowJo software. Each experiment was replicated 3 times.

### Western Blot (WB)

Tumor tissues were lysed using radioimmunoprecipitation assay (RIPA) buffer, supplemented with a protease inhibitor cocktail (Thermo Fisher Scientific, USA). Proteins were isolated by SDS-PAGE gel and transferred onto polyvinylidene fluoride (PVDF) membranes (Millipore, USA). Following blocking with 5% skim milk, the membranes were maintained overnight at 4°°C with primary antibodies. After washing the membranes, they were kept with a secondary antibody labeled with horseradish peroxidase at room temperature for 1 hour. Protein bands were detected using an ECL reagent kit (Pierce Biotechnology, USA) and a fluorescence and chemiluminescence imaging system (Clinx, China). Primary antibodies used included rabbit anti-*IFN-γ*, *IL-2*, *PD-L1* (Thermo Fisher Scientific, USA), and* GAPDH* (Abcam, UK). The secondary antibody was goat anti-rabbit IgG H&L (HRP) (Abcam, UK). Each experiment was repeated 3 times.

### Statistical Analysis

Student’s* t*-test was employed to assess data between two groups, while one-way analysis of variance (ANOVA) was used to evaluate data among 3 or more groups. Statistical significance was established with a threshold *P*-value of less than .05. All statistical analyses were conducted using GraphPad 8.0 software (GraphPad Software, La Jolla, USA).

## Results

### Correlation Between Gut Microbiota and Immune Infiltration in CRC Patients

**Figure 1.**
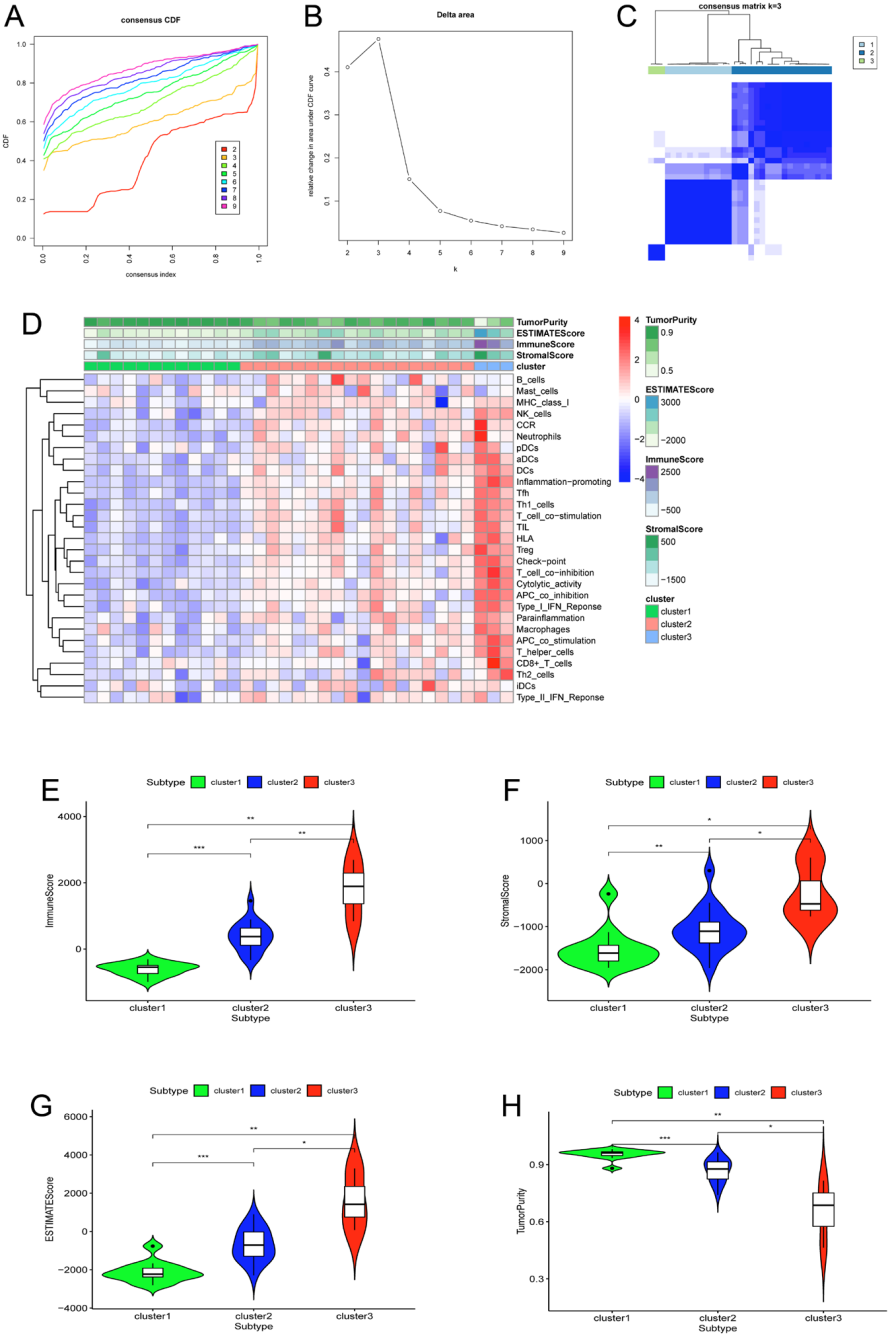
Correlation of gut microbiota with immune infiltration in CRC patients. A: Consensus clustering cumulative distribution function curve. B: CDF delta area curve. C: Heatmap showing the clustering of CRC patients into three subtypes. D: Heatmap of ssGSEA and ESTIMATE analysis results in different immune subtypes. E: Immune score. F: Stromal score. G: ESTIMATE score. H: Tumor purity. * indicates *P*< .05.

From the results of ssGSEA analysis, a consensus clustering analysis was performed on 33 CRC samples. Using the cumulative distribution function (CDF) (Figure 1A) and the CDF delta area curve (Figure 1B), the 33 clinical information-enriched cancer samples were put into 3 subtypes (Figure 1C). To validate the reliability of the 3 immune subtypes, Stromal Score, Immune Score, ESTIMATE Score, and Tumor Purity were calculated using the ESTIMATE algorithm based on the gene expression profiles of CRC patients. In contrast to the cohort characterized by low immune cell infiltration, the high immune cell infiltration group exhibited diminished tumor purity but elevated overall ESTIMATE Score, Immune Score, and Stromal Score. Cluster 1 (Low immune), cluster 2 (Middle immune), and cluster 3 (High immune) consisted of 12 samples, 18 samples, and 3 samples, respectively (Figure 1D). The boxplot results unveiled noteworthy disparities in the Stromal Score, Immune Score, ESTIMATE Score, and Tumor Purity across the 3 subtypes (Figure 1E-H).

### Taxonomic Features Similarities and Disparities in Gut Microbiota of CRC Individuals with Different Immune Subtypes

**Figure 2.**
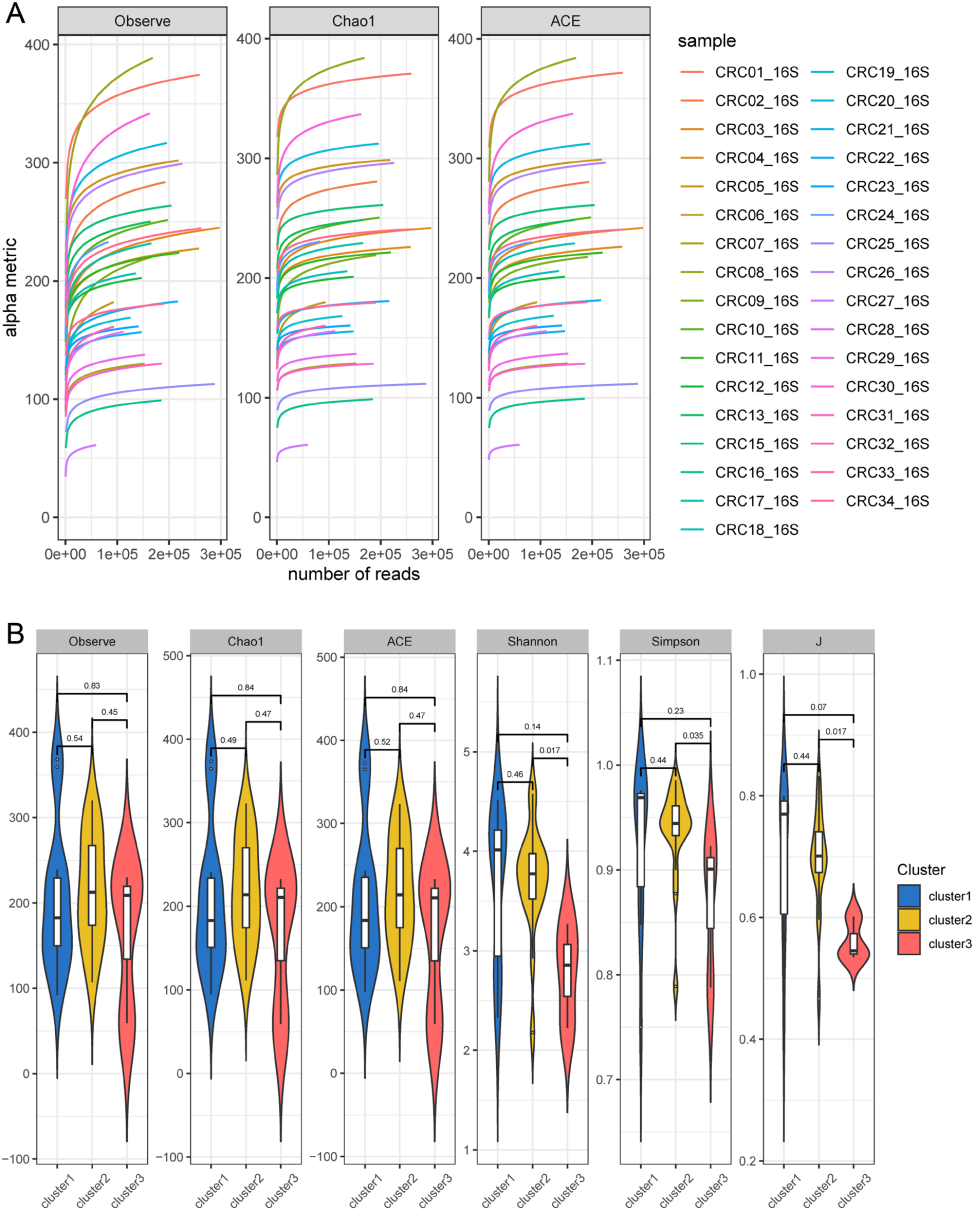
Taxonomic characteristics of gut microbiota in different immune subtypes of CRC patients. A: Sparse curve of gut microbiota in CRC patients. B: Comparison of Alpha diversity of gut microbiota in different immune subtypes of CRC patients.

We characterized the gut microbiota of 33 CRC individuals. After Illumina sequencing, a total of 7087 522 high-throughput sequence data were generated, with the maximum sequencing reads for a single sample being 356 637, the minimum being 69 390, and a median of 210 845. Following quality control steps, feature tables and feature sequences (sequences.fasta) were obtained. Based on the sparse curve, sequencing depth reached a plateau after reaching a minimum of 59 532 sequence reads for each sample (Figure 2A). A total of 2765 features were included for subsequent analysis after rarefying the sample reads to 59 532. Various metrics, including Chao1, ACE, richness indices, Jaccard index (J), observed Operational Taxonomic Units (OTUs), and Shannon index, were used to assess taxonomic and functional α-diversity. No significant differences in observed OTUs, Chao1, and ACE indices were observed between different subtypes. However, significant differences were observed in the Shannon index, Simpson index, and J index between cluster 2 and cluster 3, as well as the J index between cluster 1 and cluster 3 (Figure 2B). These findings suggested that different immune subtypes may exhibit minimal differences in species richness but significant differences in evenness, particularly in terms of microbiome evenness between cluster 2 and cluster 3 CRC patients.

**Figure 3.**
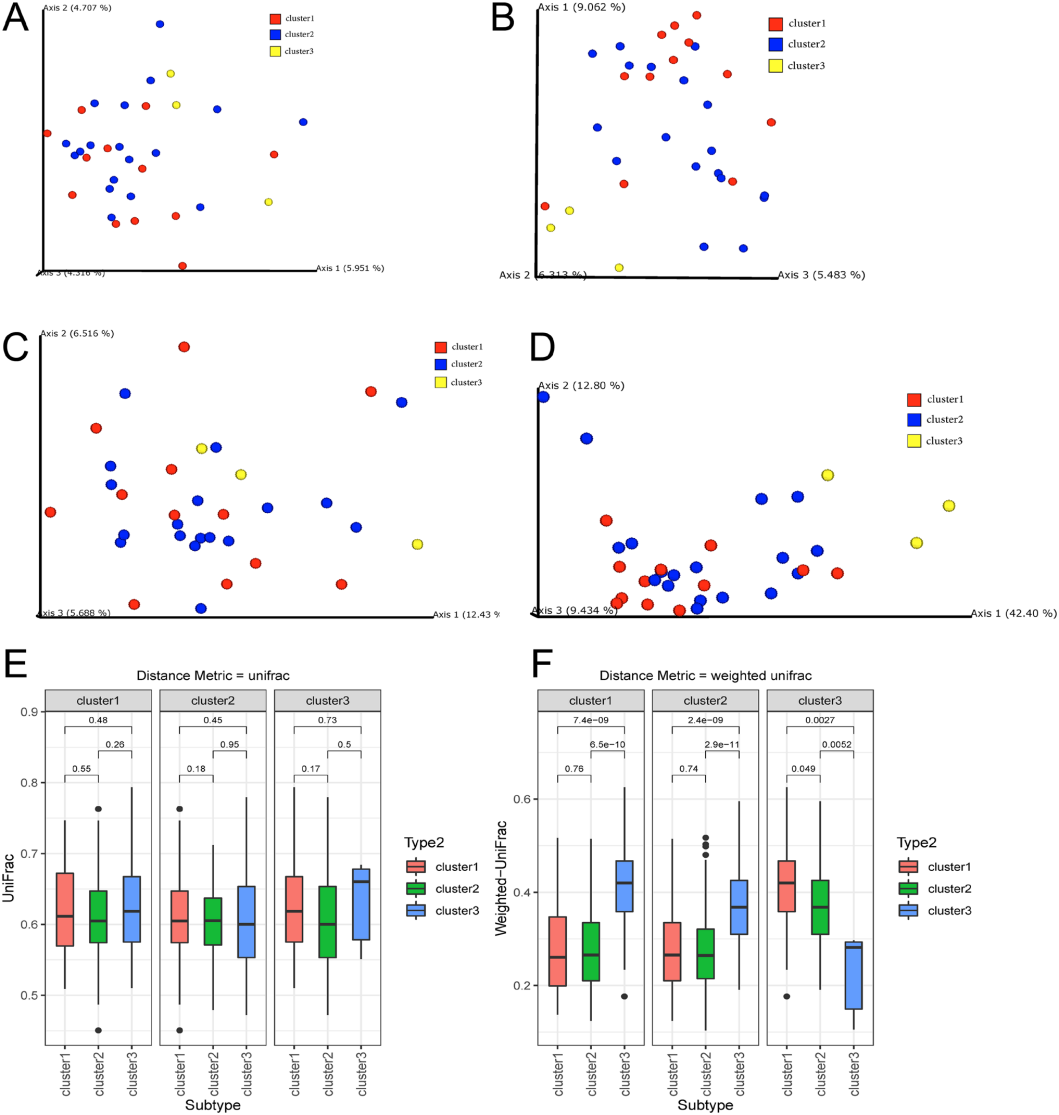
Beta diversity indices. A: Based on the Jaccard distance metric. B: Based on Bray-Curtis distance metric. C: Based on unweighted UniFrac distance metric. D: Based on weighted UniFrac distance metric. E: Differential analysis based on unweighted UniFrac distance metric. F: Differential analysis based on weighted UniFrac distance metric.

We further explored the β-diversity indices to examine potential differences in gut microbiota composition among CRC patients with different subtypes. Principal Coordinates Analysis (PCoA) results revealed that there were no significant disparities in gut microbiota community structure between different subtypes, as measured by Jaccard distance and unweighted UniFrac distance. However, significant differences were observed in Bray-Curtis distance and weighted UniFrac distance between the cluster 3 subtype, and the other 2 subtypes (Figure 3A-D). Statistical analysis based on Wilcoxon tests supported these findings (Figure 3E and  F). These results suggested that while species composition in gut microbiota may not significantly differ between different immune subtypes of CRC patients, there were significant differences in microbial community abundance.

**Figure 4.**
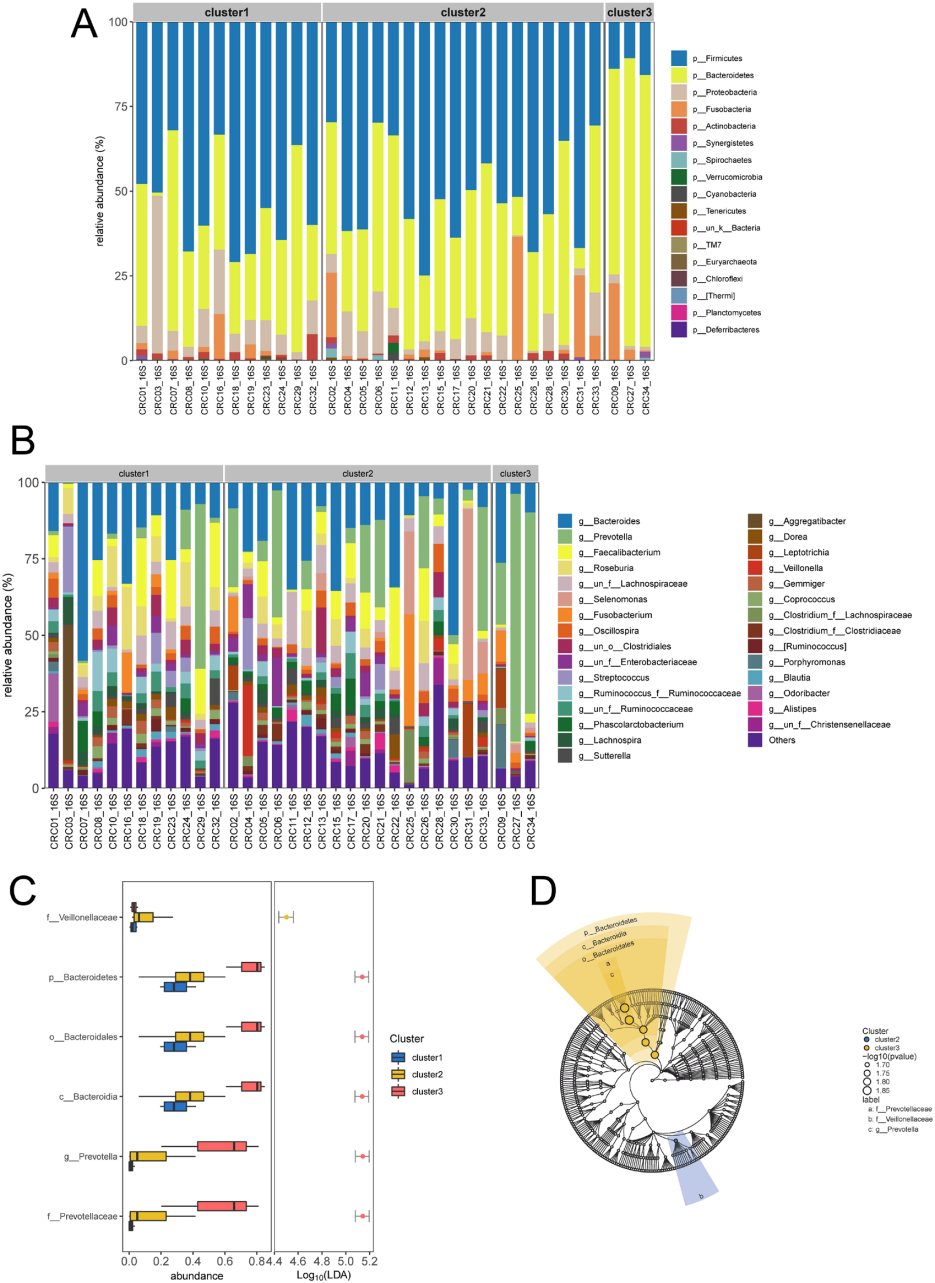
Correlation of gut microbiota abundance with immune components and immune activity gene sets in ssGSEA. A: Bar chart showing the abundance of gut microbiota at the phylum level in different immune subtypes of CRC patients. B: Bar chart showing the abundance of gut microbiota at the genus level in different immune subtypes of CRC patients. C: Box plot of differential species in different immune subtypes of CRC patients. D: A phylogenetic tree of differential species.

At the phylum level, a total of 17 phyla were observed (Figure 4A), with dominant phyla including *Firmicutes*,* Bacteroidetes*, *Proteobacteria*, and* Fusobacteria*. The results indicated that the gut microbiota of cluster 1 and cluster 2 subtypes of CRC patients were predominantly composed of *Firmicutes* and *Bacteroidetes*, whereas cluster 3 was primarily characterized by *Bacteroidetes*, with a significant decrease in *Firmicutes* abundance. At the genus level, dominant genera included *Bacteroides*, *Prevotella*, *Faecalibacterium*, and *Roseburia* (Figure 4B). Notably, the gut microbiota of cluster 3 subtype patients was primarily enriched with *Prevotella*, while the abundances of *Faecalibacterium* and *Roseburia* were significantly lower compared to cluster 1 and cluster 2 subtypes. Analysis of differentially abundant species within the subtypes revealed significant enrichment of members of the *Veillonellaceae* family in the gut microbiota of cluster 2 subtype individuals and enrichment of members of the *Prevotellaceae* family in the gut microbiota of cluster 3 subtype individuals (Figure 4C and  D), highlighting the pivotal role of *Prevotellaceae* abundance in the gut microbiota of CRC individuals.

### Relationship Between *Prevotellaceae* and Immune-related Genes in CRC

**Figure 5.**
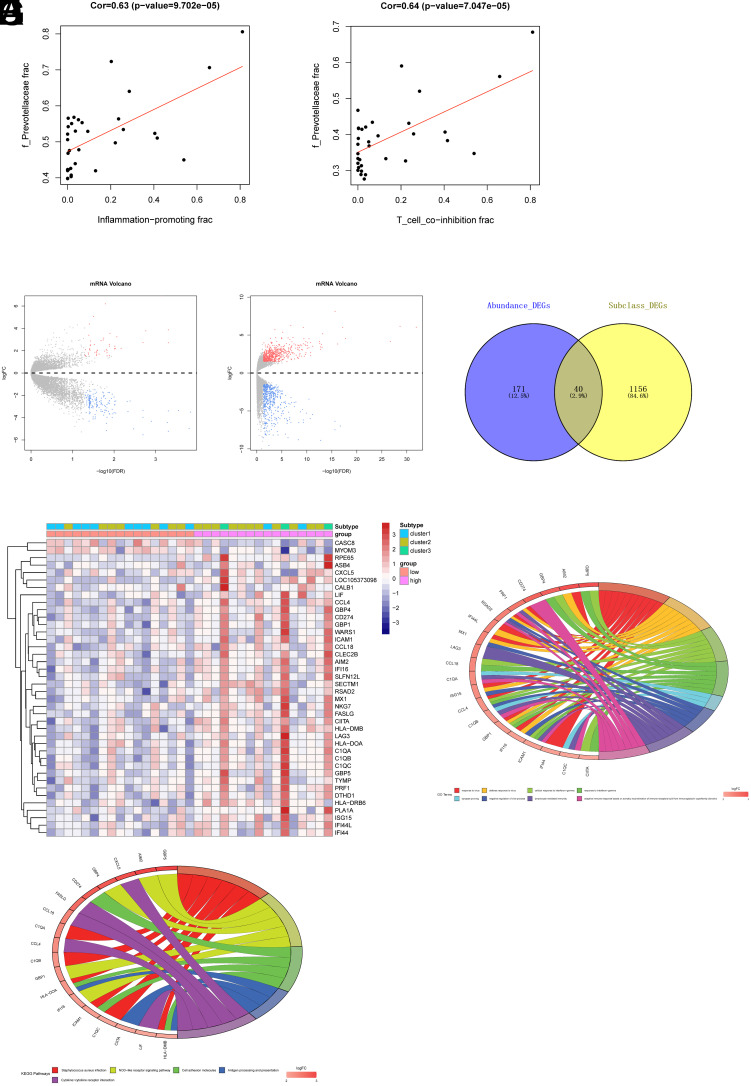
Relationship between *Prevotellaceae* and immune-related genes in CRC. A: Pearson correlation analysis of *Prevotellaceae* with inflammation-promoting. B: Pearson correlation analysis of *Prevotellaceae* with T_cell_co-inhibition. C: Volcano plot of differential mRNA in CRC patients’ gut microbiota with high and low *Prevotellaceae* abundance. D: Volcano plot of differential mRNA in different subtypes of CRC patients. E: Venn diagram of differential genes related to *Prevotellaceae* abundance and immune. F: Heatmap of differential gene expression related to both *Prevotellaceae* abundance and immune. G: GO enrichment analysis results of differential genes related to both *Prevotellaceae* abundance and immune. H: KEGG enrichment analysis results of differential genes related to both *Prevotellaceae* abundance and immune.

In the previous analysis, significant differences at the family level were identified for *Veillonellaceae* and *Prevotellaceae*. Correlation analysis was performed between these 2 groups of bacteria and immune components and activities in CRC patients. The results indicated that *Veillonellaceae* showed no correlation with inflammation-promoting and T_cell_co-inhibition. In contrast, *Prevotellaceae* exhibited significant correlations with various immune components and immune activities, with a strong association with inflammation-promoting and T_cell_co-inhibition (Figure 5A and  B).

*Prevotellaceae* was the most significantly different group among CRC patient subtypes, and its strong correlation with the immune system was observed. Here, we categorized CRC patient samples into high and low-abundance groups based on the median abundance of *Prevotellaceae* in the gut. Then, differential gene expression analysis was conducted on these 2 groups. This analysis resulted in a gene set (Abundance_DEGs) containing 211 DEGs, including 163 upregulated and 48 downregulated genes (Figure 5C). As shown in previous analyses, the gut microbiota community structures of cluster 1 and cluster 2 subtypes were relatively similar, and they both differed significantly from the cluster 3 subtype. Therefore, in this analysis, cluster 1 and cluster 2 subtypes were merged into subclass A, while the cluster 3 subtype was designated as subclass B. Gene differential expression analysis was then carried out for subclass A and subclass B. This analysis yielded a gene set (Subclass_DEGs) comprising 1196 DEGs, with 671 upregulated and 525 downregulated genes (Figure 5D). The intersection of Abundance_DEGs and Subclass_DEGs resulted in a set of 40 DEGs that were associated with both *Prevotellaceae* gut microbiota abundance and immune functions (Figure 5E). A heatmap was subsequently employed to visualize the expression patterns of these 40 different genes across different groups (Figure 5F).

The 40 DEGs were subjected to Gene Ontology (GO) and Kyoto Encyclopedia of Genes and Genomes (KEGG) functional enrichment analyses. The GO analysis revealed significant enrichment of these genes in biological functions related to “response to virus,” “cellular response to interferon-gamma,” “lymphocyte-mediated immunity,” “adaptive immune response based on somatic recombination of immune receptors built from immunoglobulin superfamily domains,” and “negative regulation of viral process” (Figure 5G). The KEGG analysis demonstrated significant enrichment of these genes in signaling pathways such as “*Staphylococcus aureus* infection,” “NOD-like receptor signaling pathway,” “Cytokine-cytokine receptor interaction,” and “Cell adhesion molecules” (Figure 5H). This denoted that *Prevotellaceae* exerted a regulatory role across multiple signaling pathways, influencing the gut microbiota composition in CRC patients.

### *Prevotellaceae* Adjuvant Anti-PD-L1 Immunotherapy

**Figure 6.**
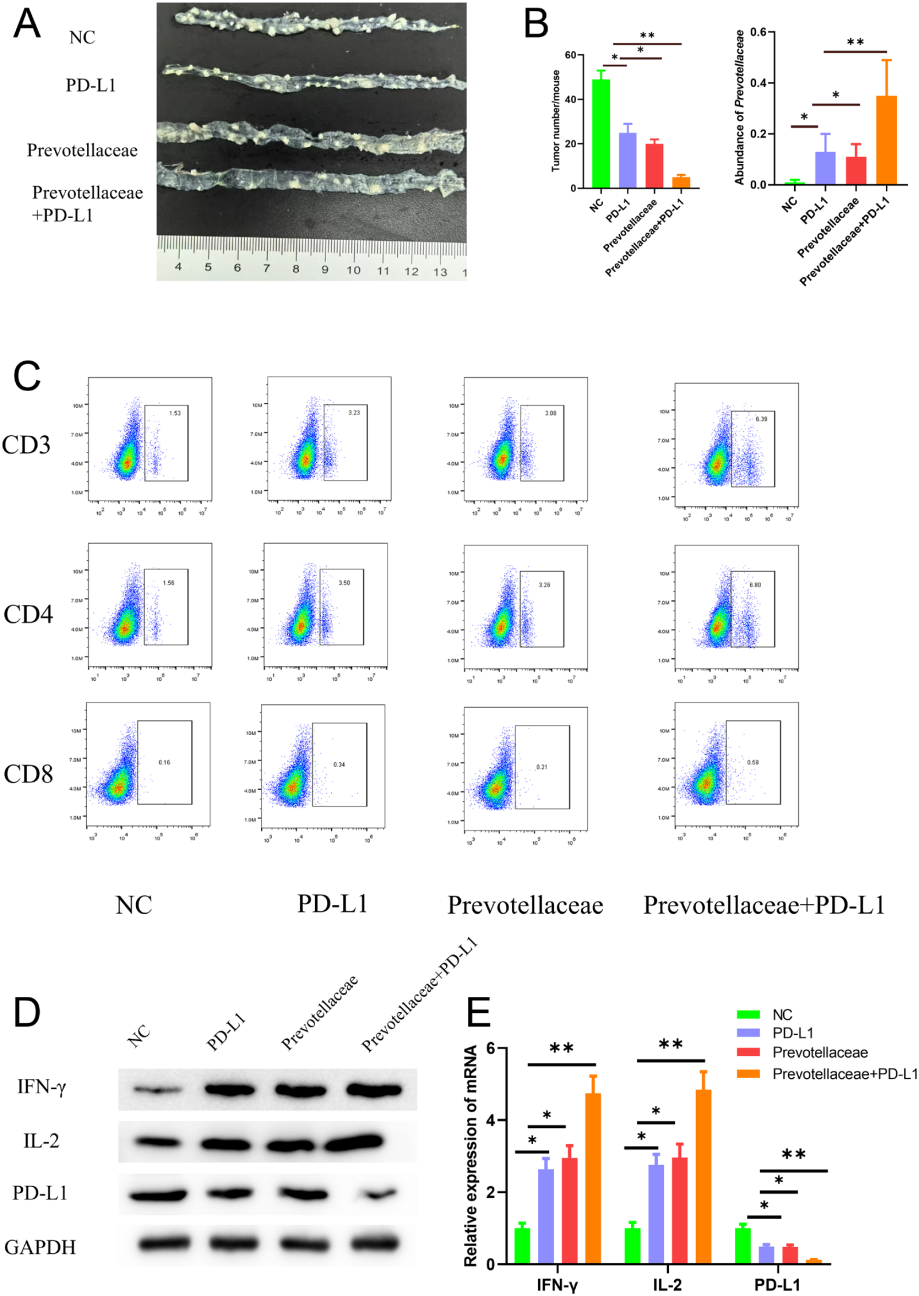
*Prevotellaceae* assists anti-PD-L1 immunotherapy. A: Observation of tumor formation in colon tissues of mice at the end of treatment. B: Analysis of *Prevotellaceae* abundance. C: Flow cytometry analysis of immune cell infiltration in tumor tissues. D-E: Detection of *IFN-γ*, *IL-2*, and *PD-L1* expression in tumor tissues by WB and qPCR. * indicates *P*< .05.

In this study, healthy male C57BL/6 mice aged 5-6 weeks were used to establish CRC mouse models by the AOM/DSS method, and they were set as the control group. The experimental group consisted of 3 groups: mice receiving anti-PD-L1 immunotherapy (group PD-L1), mice orally fed with *Prevotellaceae* (group *Prevotellaceae*), and mice orally fed with *Prevotellaceae* followed by anti-PD-L1 immunotherapy. (Group *Prevotellaceae*+PD-L1). Two weeks after the treatment, the mice were euthanized, and colon tissues were collected to assess tumor formation. The outcomes revealed that, compared to the control group, both group PD-L1 and group *Prevotellaceae* exhibited a significant reduction in the number of colon tumors. Notably, group *Prevotellaceae*+PD-L1 showed a further significant decrease in tumor numbers (Figure 6A). Fecal samples were collected from the mice, and 16S sequencing was performed to analyze the abundance of *Prevotellaceae* in the gut. The analysis indicated that, in comparison to the control group, both group PD-L1 and group *Prevotellaceae* had significantly higher *Prevotellaceae* abundance in their feces. Furthermore, group *Prevotellaceae*+PD-L1 exhibited a further increase in *Prevotellaceae* abundance (Figure 6B). Immune cell infiltration within tumor tissues was assessed using flow cytometry, with results displaying that the levels of CD3, CD4, and CD8 immune cells within the tumor tissues were notably elevated in both group PD-L1 and group *Prevotellaceae*. Importantly, group *Prevotellaceae*+PD-L1 exhibited a further increase in immune cell infiltration levels within tumor tissues (Figure 6C).

Subsequently, WB and qPCR were used to measure the expression of *IFN-γ*, *IL-2*, and *PD-L1* in tumor tissues. The findings revealed a significant upregulation of *IFN-γ* and *IL-2*, coupled with a notable downregulation of *PD-L1* in both group PD-L1 and group *Prevotellaceae*. Remarkably, group *Prevotellaceae*+PD-L1 exhibited a further significant upregulation of *IFN-γ* and *IL-2*, along with a further significant downregulation of *PD-L1* (Figure 6D and  E). These findings indicated that *Prevotellaceae* effectively enhanced the efficacy of anti-PD-L1 immunotherapy.

## Discussion

Colorectal cancer is a prevalent malignancy within the gastrointestinal tract. Existing treatments for CRC include early tumor surgery and, for advanced patients, chemotherapy or radiation therapy. In recent years, as immunotherapy has come to the forefront, ICB strategies have been applied to CRC treatment.^18,19^ For instance, PD-1, PD-L1, and members of the CD28 superfamily of T cell regulatory factors have been identified as potential targets for immunotherapy in CRC.^20^ Recently, research has shown that the microbiome can influence the effectiveness of cancer immunotherapy. For example, Gao and colleagues^21^ found that high levels of *Fusobacterium nucleatum* were associated with improved treatment responses to PD-1 blockade in CRC patients. *F. nucleatum* enhanced the anti-tumor effects of PD-L1 blockade in mouse models of CRC, leading to extended survival.

The gut microbiota, including families like *Rikenellaceae* and *Ruminococcaceae*, along with their metabolic byproducts such as short-chain fatty acids (SCFAs), play pivotal roles in maintaining intestinal barrier integrity and homeostasis.^22^ For instance, the *Rikenellaceae* family, belonging to the *Bacteroidales* order, has been associated with resistance to immune-related colitis induced by CTLA-4 inhibitors,^23^ and a reduction in taxa that produce butyrate has been linked to increased systemic inflammation and atherosclerosis.^24^ Conversely, the expansion of certain bacteria, particularly *Shigella* species, has been positively correlated with gastrointestinal toxicity induced by ICIs.^25^

In this study, through bioinformatics analysis, we observed that, in comparison to the low immune cell infiltration group, the high immune cell infiltration group exhibited reduced tumor purity. Notably, the 3 subtypes displayed significant disparities in Stromal Score, Immune Score, ESTIMATE Score, and Tumor Purity. Cluster 2 and cluster 3 CRC patients exhibited a marked difference in microbial community evenness. Notably, members of the *Prevotellaceae* family were significantly enriched in cluster 3 CRC patients’ gut microbiota. In conclusion, a higher abundance of *Prevotellaceae* in CRC is associated with increased immune infiltration. A previous study suggested that enrichment of *Prevotellaceae*, *Ruminococcaceae*, and *Lachnospiraceae* is associated with a favorable response to PD-1/PD-L1 therapy.^26^ Our *in* *vivo* experiments confirmed that a high abundance of *Prevotellaceae* enhanced the efficacy of anti-PD-L1 tumor therapy. Therefore, therapeutic agents that modulate the gut microbiota, such as probiotics, have the potential to be one of the most effective approaches to combat CRC.

The findings of our study demonstrated for the first time that *Prevotellaceae* abundance influenced the efficacy of CRC anti-PD-L1 immunotherapy, confirming that a high abundance of *Prevotellaceae* enhanced the efficacy of anti-PD-L1 tumor therapy. Nevertheless, it should be emphasized that our investigation was conducted using murine models, and the extent to which *Prevotellaceae* abundance influences the efficacy of PD-1/PD-L1 inhibitors in human cancer remains a topic that requires further exploration. Further validation of these results in human subjects is needed in the future. Viewed in toto, our results indicated that alterations in the gut microbiota impacted tumor immunotherapy. Therefore, the modulation of the gut microbiome, such as increasing *Prevotellaceae* abundance, could serve as a novel approach for CRC treatment.

## Figures and Tables

**Table 1.** Primers for qPCR

Gene	Sequence	
*IFN-γ*	Forward Primer	5’-GCATTCCAGTTGCTGCCTACT-3’
	Reverse Primer	5’-ACCAGGCATGAGAAGAAATGCT-3’
*IL-2*	Forward Primer	5’-CCTCAACTCCTGCCACAATGT-3’
	Reverse Primer	5’-TGCGACAAGTACAAGCGTCAGT-3’
*PD-L1*	Forward Primer	5’-GTGGCATCCAAGATACAAACTCAA-3’
	Reverse Primer	5’-TCCTTCCTCTTGTCACGCTCA-3’
*GAPDH*	Forward Primer	5’-AAGAAGGTGGTGAAGCAGGC-3’
	Reverse Primer	5’-TCCACCACCCAGTTGCTGTA-3’

## Data Availability

The data that support the findings of this study are available from the corresponding author.
